# Predicting the polyspecificity of aminoacyl-tRNA synthetase for non-canonical amino acids using molecular dynamics simulation and MM/PBSA

**DOI:** 10.1371/journal.pone.0316907

**Published:** 2025-01-10

**Authors:** Dongheon Lee, Jong-il Choi

**Affiliations:** Department of Biotechnology and Bioengineering, Chonnam National University, Gwangju, Republic of Korea; National Institute of Health, INDIA

## Abstract

With the advancement of genetic code expansion, the field is progressing towards incorporating multiple non-canonical amino acids (ncAAs). The specificity of aminoacyl-tRNA synthetases (aaRSs) towards ncAAs is a critical factor, as engineered aaRSs frequently show polyspecificity, complicating the precise incorporation of multiple ncAAs. To address this challenge, predicting binding affinity can be beneficial. In this study, we expressed sfGFP using an orthogonal aaRS/tRNA pair with 4-Azido-L-phenylalanine (AzF) and another four different ncAAs. The experimental results showed specificity with O-Methyl-L-tyrosine as well as AzF, and these results were compared with computational predictions. We constructed a mutant aaRS structure specific for AzF through homology modelling and conducted docking studies with tyrosine and five ncAAs, followed by molecular dynamics simulations. The binding affinity was calculated using the molecular mechanics/Poisson–Boltzmann surface area, focusing on nonpolar solvation terms. While the analysis is based on the incorporation of limited number of ncAAs, the cavity and dispersion term method showed consistency with experimental data, highlighting its potential utility compared to the surface area term method. These findings enhance understanding of the ncAA specificity of aaRS in relation to computer simulations and energy calculations, which can be utilized to rationally design or predict the specificity of aaRS.

## Introduction

Genetic code expansion (GCE) enables the site-specific incorporation of non-canonical amino acids (ncAAs) into proteins, enhancing chemical functions, ligand binding, fluorescence, photocaging, and cross-linking [1]. Recently, there have been attempts to incorporate multiple ncAAs into proteins to introduce various functional groups [2, 3]. As the number of ncAAs increases, additional orthogonal aminoacyl-tRNA synthetase (aaRS) and tRNA pairs are needed. Pairs of aaRS/tRNA have been imported from phylogenetically distant organisms to achieve orthogonality, such as *Methanococcus jannaschii* TyrRS/tRNA^Tyr^, *Methanosarcina barkeri* PyrRS/tRNA^Pyr^, and *Methanosarcina mazei* PylRS/tRNA^Pyl^. Many aaRS/tRNA pairs have been developed to expand the tools for GCE [4–8], and efforts are ongoing to develop mutually orthogonal aaRS/tRNA pairs, such as quintuply orthogonal PyrRS/tRNA^Pyl^ pairs [9].

Furthermore, achieving the production of proteins with multiple ncAAs in the realm of GCE requires the orthogonality of genetic codes, ncAAs, aaRS/tRNA pairs, and ribosomes, ensuring compatibility with native cellular components as well as exogenous factors. It is essential to facilitate gene expression and translation processes without cross-reactivity or pleiotropic effects [10]. Establishing these conditions is a key factor in producing intended proteins incorporating multiple ncAAs simultaneously and accurately. However, many engineered orthogonal aaRSs have the property of being polyspecific, meaning they recognize multiple ncAAs [11–13], and the orthogonality between ncAAs and aaRS is sometimes overlooked because a small number of ncAAs have been incorporated into a single protein. The orthogonality of aaRS in recognizing ncAAs will be increasingly important as the number of ncAAs to be incorporated simultaneously into a single protein increases. However, the polyspecificity is a significant drawback in producing proteins with multiple ncAAs as it disrupts specific incorporation. Therefore, predicting the recognition of ncAAs by aaRS could be helpful in producing recombinant proteins utilizing multiple ncAAs.

On the other hand, the rational design of the active site, or interpreting the relationship between aaRSs and ncAAs is often performed using computational approaches. For instance, a structure-based design approach was used to engineer *M*. *jannaschii* TyrRS to specifically incorporate O-Methyl-L-tyrosine through computational predictions and molecular dynamics (MD) simulations [14]. Similarly, rational design and computational modelling were employed to create a specific aaRS for 4-Acetyl-L-phenylalanine, enhancing its selectivity by predicting and validating mutations that improve binding affinity [15]. Additionally, favourable and unfavourable aaRS–amino acid complexes were clustered using the Molecular Mechanics/Poisson–Boltzmann Surface Area (MM/PBSA) method, which can be performed either following MD simulations or directly [16]. In contrast to these studies that focused on specific ncAAs or distinguishing favourable ones from unfavourable ncAAs, we aimed to arrange and validate the recognition of ncAAs in relation to binding affinity derived from the polyspecificity of aaRSs using both experimental and computational methods.

In this study, we incorporated five ncAAs into green fluorescent protein (GFP) containing an amber codon and calculated normalized fluorescence levels to estimate the activity of aaRS according to ncAAs. For the *in silico* research, we used homology modelling to predict the aaRS structure and performed docking studies of amino acids. Amino acid–aaRS complexes obtained from the docking studies were subjected to MD simulations to optimize the binding pose in the active site and interactions between the ligand and receptor. The binding free energy was calculated using two different methods for nonpolar solvation energy within the MM/PBSA, based on simulation trajectories, and compared with *in vivo* results. Subsequently, analysis of the amino acid–aaRS complexes allowed for the identification of the key factors contributing to amino acid recognition.

## Materials and methods

### Strain, media, and plasmids

**Fig 1:**
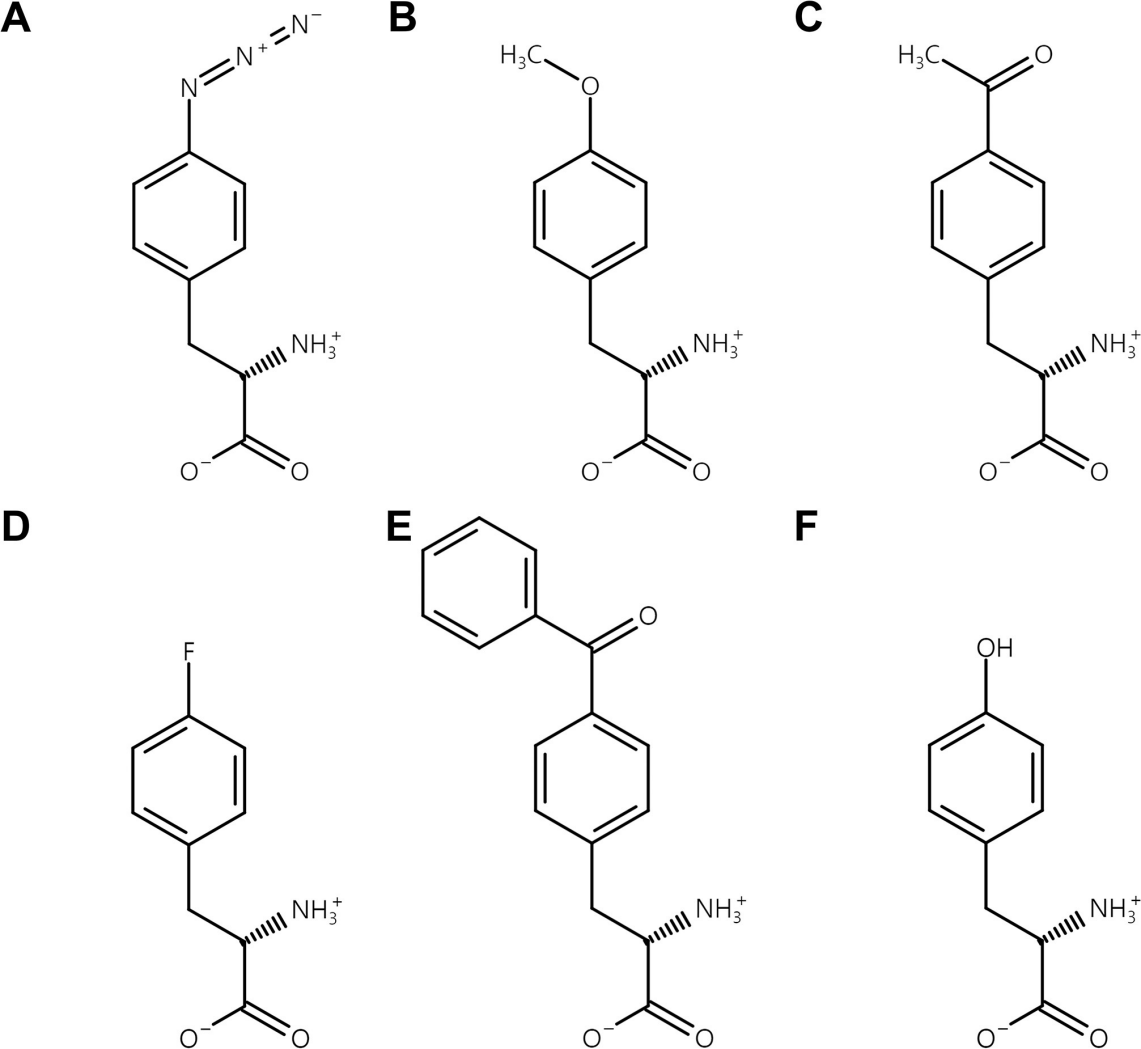
Non-canonical amino acid structures used in this study. Each amino acid was subjected to incorporation by a mutant aaRS derived from *M*. *jannaschii* TyrRS, which was initially developed for AzF. (A) 4-Azido-L-phenylalanine (AzF). (B) O-Methyl-L-tyrosine (OMY). (C) 4-Acetyl-L-phenylalanine (AcF). (D) 4-Fluoro-L-phenylalanine (pFF). (E) 4-Benzoyl-l-phenylalanine (BpF). (F) L-tyrosine (Tyr).

*Escherichia coli* DH10B [F–*mcrA* Δ*(mrr-hsdRMS-mcrBC)* φ80*lacZ*ΔM15 Δ*lacX74 recA1 endA1 araD139* Δ*(ara-leu)*7697 *galU galK λ–rpsL(*Str^R^*) nupG*] (Invitrogen, Waltham, MA) was used for recombinant protein expression. Cell cultures were grown in LB media. A total of five ncAAs were used for ncAA incorporation: 4-Azido-L-phenylalanine (AzF), O-Methyl-L-tyrosine (OMY), 4-Acetyl-L-phenylalanine (AcF), 4-Fluoro-L-phenylalanine (pFF), and 4-Benzoyl-l-phenylalanine (BpF) (Fig 1). The pEVOL-pAzF-C1 plasmid was used for the expression of orthogonal aaRS and tRNA pair under the control of the araBAD and proK promoters, respectively [17]. The pSEVA631pt-sfGFP204amb plasmid was used for the expression of superfolder green fluorescent protein (sfGFP) containing an amber codon at position 204 under the control of tac promoter [17].The working antibiotics concentrations were 50 μg/mL chloramphenicol for pEVOL-pAzF-C1 and 50 μg/mL gentamicin for pSEVA631pt-sfGFP204amb.

### Reporter protein expression and fluorescence measurements

For the seed culture, a fresh single colony of *E*. *coli* DH10B (pEVOL-pAzF-C1, pSEVA631pt-sfGFP204amb) was inoculated in 4 mL of LB broth supplemented with antibiotics and grown overnight at 37°C and 250 rpm. sfGFP was expressed by inoculating the seed culture at a 1:50 ratio in 4 mL of LB broth supplemented with antibiotics, 0.2% L-(+)-arabinose, 0.5 mM isopropyl β-D-1-thiogalactopyranoside (IPTG), and 0.5 mM ncAA, and culturing for 9 hours at 37°C and 250 rpm. Optical density (600 nm) and fluorescence (excitation 485 nm, emission 528 nm) were measured using a SpectraMax iD3 (Molecular Devices, San Jose, CA). The normalized fluorescence intensity was calculated by dividing the fluorescence value by the optical density. The relative normalized fluorescence was calculated by dividing the fluorescence values (excitation at 485 nm, emission at 528 nm) by the optical density at 600 nm, with AzF set as 100%.

### Homology modelling and molecular docking

The amino acid sequence of aaRS from pEVOL-pAzF-C1 was submitted to SWISS-MODEL [18]. Using the crystal structure of *M*. *jannaschii* TyrRS (PDB 1J1U) [19], the tertiary structure prediction of aaRS, which was optimized for the incorporation of AzF [20], was conducted. AutoDock Vina 1.2.0 [21] was used for docking ligands into the aaRS structure. The ligand structures of five ncAAs and tyrosine were obtained from PubChem [22]. Using the crystal structure of *M*. *jannaschii* TyrRS as a reference, a 5 Å radius and a 11.25 × 11.25 × 11.25 Å^3^ cubic grid box around the substrate tyrosine position were selected as the flexible amino acid residues and ligand binding site, respectively. From the docking study, the best-scored binding pose was selected for MD simulations except in special case where the carboxyl and amino groups were oriented toward the deep pocket and the functional group faced outward. This case makes it difficult to interact with ATP for aminoacylation.

### Complex preparation and molecular dynamics simulation

The protein–ligand complex was prepared based on the results of the flexible docking study described above. The structures of the aaRSs were analysed using the H++ server [23] to estimate their protonated states in a physiological environment, and parameters were generated using ff14SB [24]. The ligand parameters were generated using the general AMBER force field 2 (gaff2) [25, 26]. The protein–ligand complexes were solvated in a truncated octahedron box, which required fewer water molecules and fit globular proteins better than cubic boxes [27]. The water box was created with a 10 Å buffer of TIP3P water [28], 1 Cl^-^ ion for system neutralization, and 0.15 mM NaCl ions.

All relaxation and MD simulations were performed using AMBER22 [29]. The simulations were performed under periodic boundary conditions (PBC). Long-range electrostatic interactions were computed using the Particle Mesh Ewald (PME) method with an 8 Å non-bonded cutoff.

The relaxation process consisted of 9 steps, each lasting 5000 ps with a time step of 1 fs, except for energy minimization. The SHAKE algorithm was utilized to constrain the covalently bonded hydrogen atoms in all steps except for energy minimization. Unlike the aaRS, which was restrained, the ligand was not restrained during the relaxation. Initial energy minimization was performed for 5000 cycles, with the first 1000 cycles using the steepest descent method and the remaining 4000 cycles using the conjugate gradient algorithm. A force constant of 100 kcal‧mol^-1^‧Å^-2^ was applied to restrain the protein atoms. The system was heated from 100 K to 310 K, and subsequently relaxed at 1 atm to equilibrate the box pressure and density. The restraint was gradually reduced to a lower force constant of 10 kcal‧mol^-1^‧Å^-2^. The system underwent another round of minimization using the same method as the initial energy minimization, but this time only the protein backbone was restrained with a force constant of 10 kcal‧mol^-1^‧Å^-2^. Then, the system was relaxed by gradually reducing the force constants on the protein backbone of 10 kcal‧mol^-1^‧Å^-2^, 1 kcal‧mol^-1^‧Å^-2^, and 0.1 kcal‧mol^-1^‧Å^-2^ over each 5000 ps. Finally, all restraints were removed in the last relaxation step. The Langevin thermostat and Berendsen barostat were used to maintain a constant system temperature and pressure, respectively.

MD simulations were performed in triplicate for 200 ns with a time step of 4 fs. After relaxation, random velocities were assigned, and unrestrained MD simulations were carried out. To reduce computational cost, hydrogen mass repartitioning and the NVT ensemble were adjusted for the increased time step [30]. Coordinates were saved every 5 ps throughout the production runs.

### MM/PBSA

MMPBSA.py in AmberTools was used to calculate the binding free energies [31, 32]. A total of 201 snapshots were analysed at 500 ps intervals throughout the 100–200 ns trajectory. The ionic strength was set to 0.15 mM, with internal and external dielectric constants set to 2 and 80, respectively [33]. The nonpolar solvation was calculated using either the SASA model (inp = 1) or the CD (cavity and dispersion) model (inp = 2). The SASA model estimates the nonpolar solvation energy as proportional to the solute SASA and is represented by:

$${\Delta G}_{np}=\gamma ∙\mathrm{SASA}+b$$ (1)

where γ is the surface tension coefficient, and b is the offset for the nonpolar free energy contribution. The CD model divides the nonpolar solvation energy into two terms, cavity and dispersion, described as:

$${\Delta G}_{np}={\Delta G}_{cav}+{\Delta G}_{disp}$$ (2)

where ΔG_cav_ is the cavity formation free energy corresponding to the solvation free energy from solute–solvent repulsive interactions and the formation of solute cavity. ΔG_disp_ is the dispersion free energy corresponding to establishing solute–solvent attractive interactions, including the solvent–solvent reorganization component [34, 35]. The radii and constants were determined based on the nonpolar solvation method following the radii study and AMBER manual [29, 36, 37]. PARSE radii [36] were used for the SASA term method, while the optimized radii by Tan & Luo [37] were used for the CD term method. Surface tension and offset were set to 0.00542 and 0.92 for the SASA term method, and to 0.0378 and −0.5692 for the CD term method [36, 37]. Other settings were kept at default values.

### Clustering and analysis

The representative structure was obtained using the density-based spatial clustering of applications with noise (DBSCAN) algorithm in the cpptraj module of AmberTools [32, 38]. A total of 80 amino acids around the substrate within 10 Å and the ligand were subjected to clustering. The representative structure of protein–ligand complex was analysed using the protein–ligand interaction profiler (PLIP) [39]. The occupancy of hydrogen bonds within a 100–200 ns trajectory was analysed with the cpptraj module in AmberTools. Structure visualization was conducted using PyMol [40].

## Results and discussion

### Experimental results

Aminoacylation is the process where an amino acid is attached to the active site, activated with ATP to form the aminoacyl-MP intermediate, and then transferred to the tRNA, releasing AMP. This process is mediated by aaRSs, which select their amino acid and tRNA substrates from disparate pools of chemically and structurally similar molecules and link them together to form charged aminoacyl-tRNA for protein synthesis [41]. To explore the polyspecificity of aaRSs, a mutant *M*. *jannaschii* TyrRS developed to incorporate AzF [20] was examined to determine whether it could incorporate other ncAAs. As AzF has an aryl azide in its functional group, four ncAAs (OMY, AcF, pFF, BpF) containing an aromatic ring in their functional groups were selected and these ncAAs had following advantages. AzF is utilized in click chemistry through azide–alkyne cycloaddition and this property can be used for conjugation, such as between albumin and drug [42]. Similarly, AcF is used in click chemistry for oxime or hydrazone reactions in bioconjugation [43, 44]. Photoactivatable property of BpF makes it ideal for studies of protein–protein interactions using photocrosslinking [45, 46]. OMY was employed for tumor imaging [47], and incorporating pFF enhanced the shelf life of enzyme activity [48].

**Fig 2:**
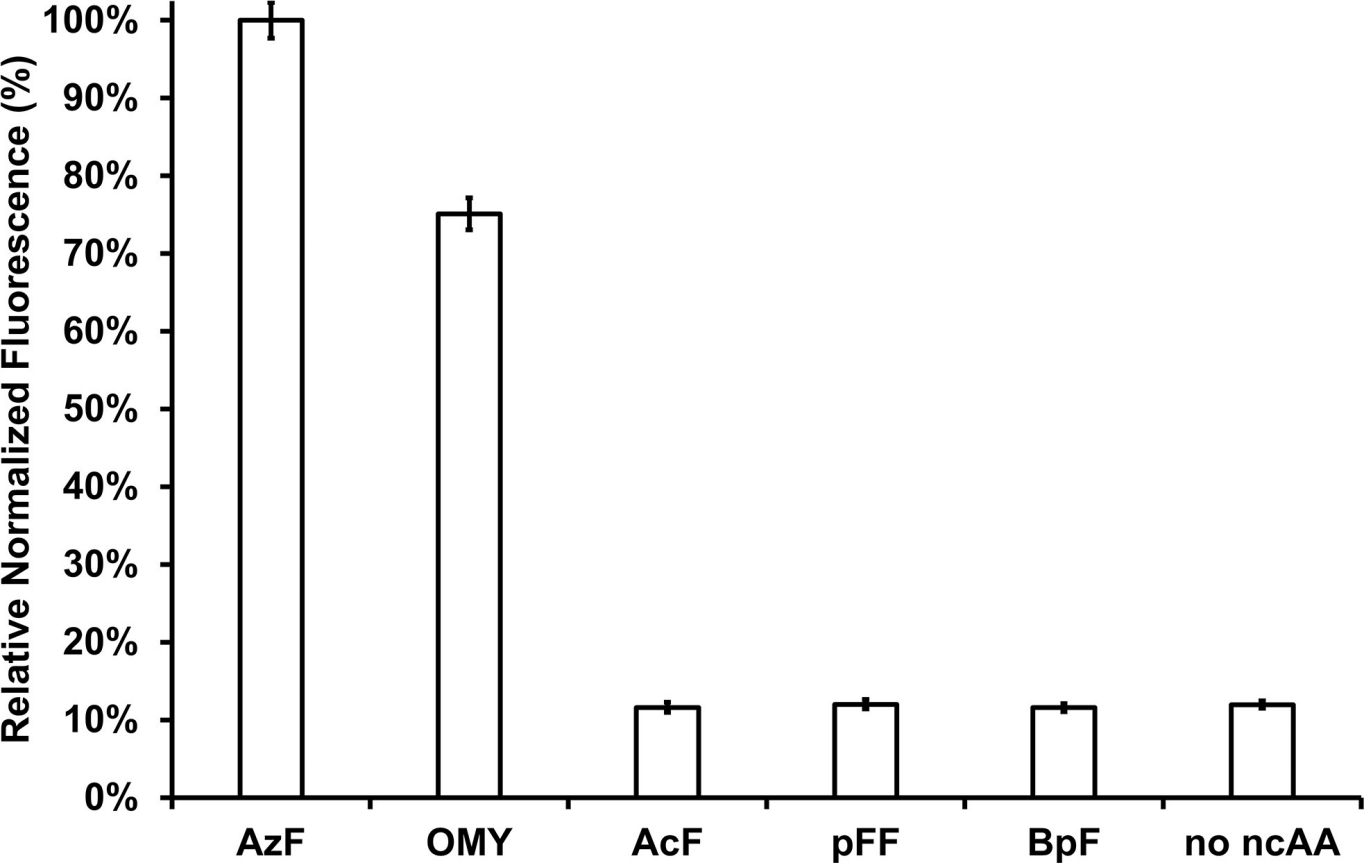
Relative normalized fluorescence levels obtained with different ncAAs. Compared to AzF, OMY showed 75% relative normalized fluorescence, while the remaining ncAAs exhibited 12%, a level similar to the fluorescence observed in the absence of any ncAA. Error bars represent the standard deviation, and all experiments were performed in triplicate.

The aaRS/tRNA pair and sfGFP were expressed with each ncAA to verify the incorporation of ncAA. As the corresponding tRNA suppresses the amber codon, sfGFP containing the amber codon in the middle of its sequence was utilized to measure activity. Without the amber suppression, the translation of sfGFP was incomplete due to truncation, resulting in a lack of fluorescence signal. Accordingly, enzyme activity was measured by calculating relative normalized fluorescence intensity using sfGFP (Fig 2). When the relative normalized fluorescence intensity of AzF was set to 100%, OMY showed 75% of relative normalized fluorescence indicating the mutated aaRS could efficiently incorporate OMY as well as AzF. In contrast, AcF, pFF, and BpF showed 12% of relative normalized fluorescence, which was identical to the value obtained in the absence of ncAAs. It was consistent with the observation that the *M*. *jannaschii* TyrRS derived aaRS/tRNA pair exhibited background expression levels in the absence of its cognate ncAA [49]. These results demonstrated that the identically mutated aaRS showed different relative normalized fluorescence levels depending on the amino acid, suggesting that the binding affinity of ncAAs affects GFP expression. Supporting this, a study reported that site mutations of a single residue interacting with ncAA results in different normalized fluorescence values, indicating that the recognition of ncAA affects GFP expression [50].

### Homology modelling and molecular docking

**Fig 3:**
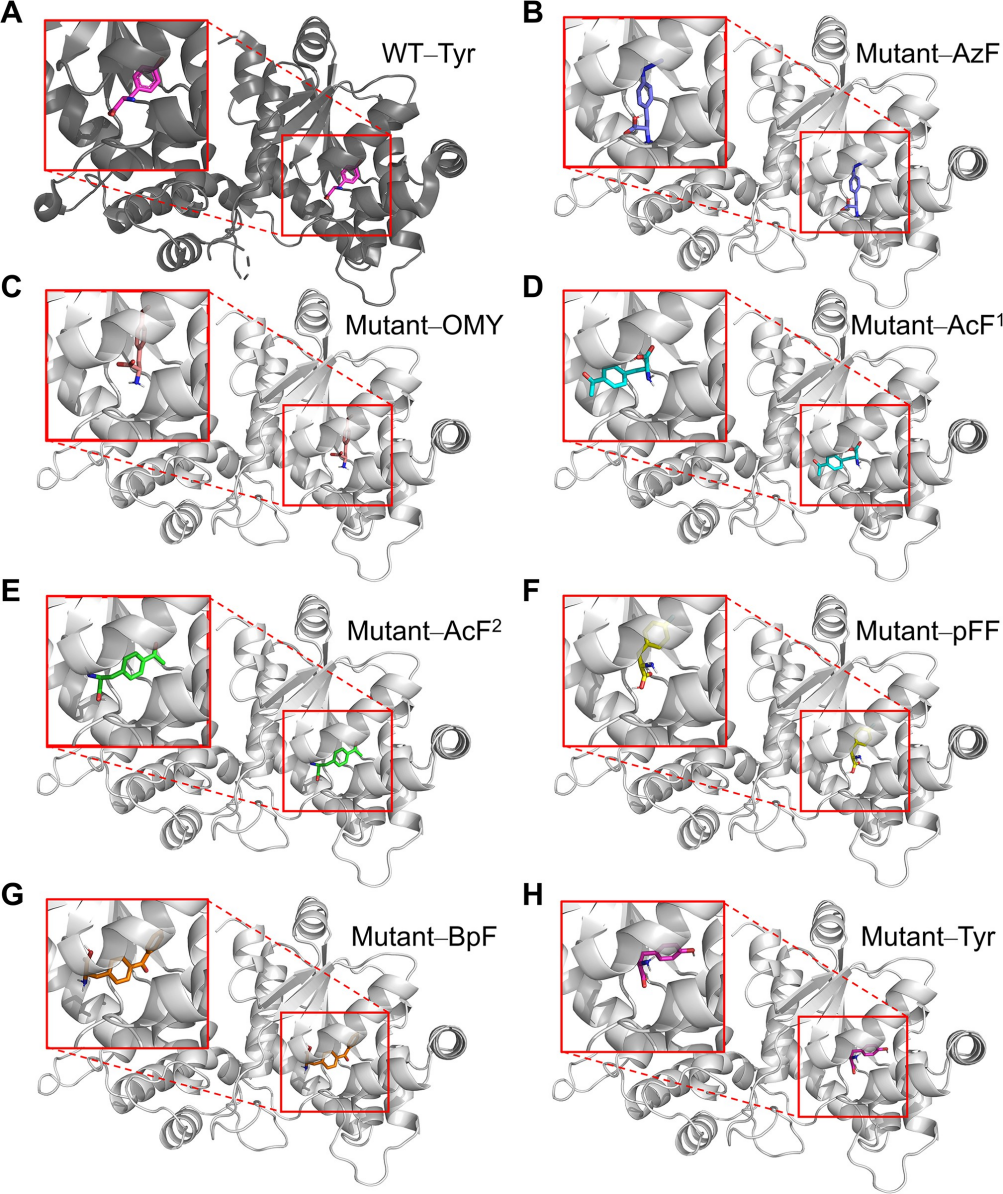
Top-scored docking poses of ncAAs. (A) Crystal structure of *M*. *jannaschii* TyrRS WT and tyrosine (PDB 1J1U), (B-H) Docking poses of mutant aaRS derived from *M*. *jannaschii* TyrRS and ncAAs. The top-scored docking pose of AcF (mutant–AcF^1^) is inverted, while the second-scored pose (mutant–AcF^2^) shows the correct orientation. *M*. *jannaschii* TyrRS WT (dark grey), mutant aaRS (light grey), tyrosine (magenta), AzF (violet), OMY (salmon), AcF (cyan or green), pFF (yellow), and BpF (orange).

Compared to the wild type of *M*. *jannaschii* TyrRS, the mutant aaRS used in this study was developed with a total of six mutations [51]. Five of these mutations—Tyr32Thr, Glu107Asn, Asp158Pro, Ile159Leu, and Leu162Gln—were introduced at the active site using site-saturation mutagenesis to recognize AzF [20]. Specifically, the Tyr32Thr and Asp158Pro mutations removed hydrogen bonds from the phenolic group of tyrosine, expanding the active site to accommodate the larger azido group. The other mutation, D286R, enhanced the recognition of tRNA anticodon [19]. The structure of the mutant aaRS was predicted using homology modelling based on the X-ray crystal structure of *M*. *jannaschii* TyrRS (PDB 1J1U) [19]. The predicted structure was directly used for the docking study as the sequence identity was 98%. Since the side chains of the active site were expected to mainly affect the binding of the substrate, the interacting poses of side chains around the substrate needed to be considered. Based on the crystal structure, amino acids within 5 Å of the substrate tyrosine position (THR32, ILE33, GLY34, PHE35, GLU36, LEU65, ALA67, HIS70, ILE137, TYR151, GLN155, PRO158, GLN173, ILE176) were treated as flexible. Flexible docking was conducted using AutoDock Vina [21]. The top-scored affinities are presented in Table 1, with additional details provided in S1 Table. The top-scored docking poses are shown in Fig 3. All amino acids, except for AcF, docked similarly to Tyr, aligning their functional groups toward the deep pocket. In contrast, the top-scoring docking pose of AcF (Fig 3D) was inverted, with the amino and carboxyl groups facing the deep pocket and the functional group positioned outward, which likely resulted in an inactive form. Therefore, the second-ranked structure (Fig 3E), which exhibited the correct orientation, was selected for further analysis. According to the docking affinity results, AzF showed the lowest energy, which corresponded to experimental data. However, other amino acids showed lower energy than OMY, which could not explain the incorporation of OMY.

**Table 1:** Results of binding affinity from docking study.

	AzF	OMY	AcF	pFF	BpF	Tyr
Affinity (kcal/mol)	−4.376	−1.791	−1.36 (−2.598)^a^	−2.23	−2.611	−2.569

^a^ Inverted docking pose

### MD simulation and MM/PBSA

**Fig 4:**
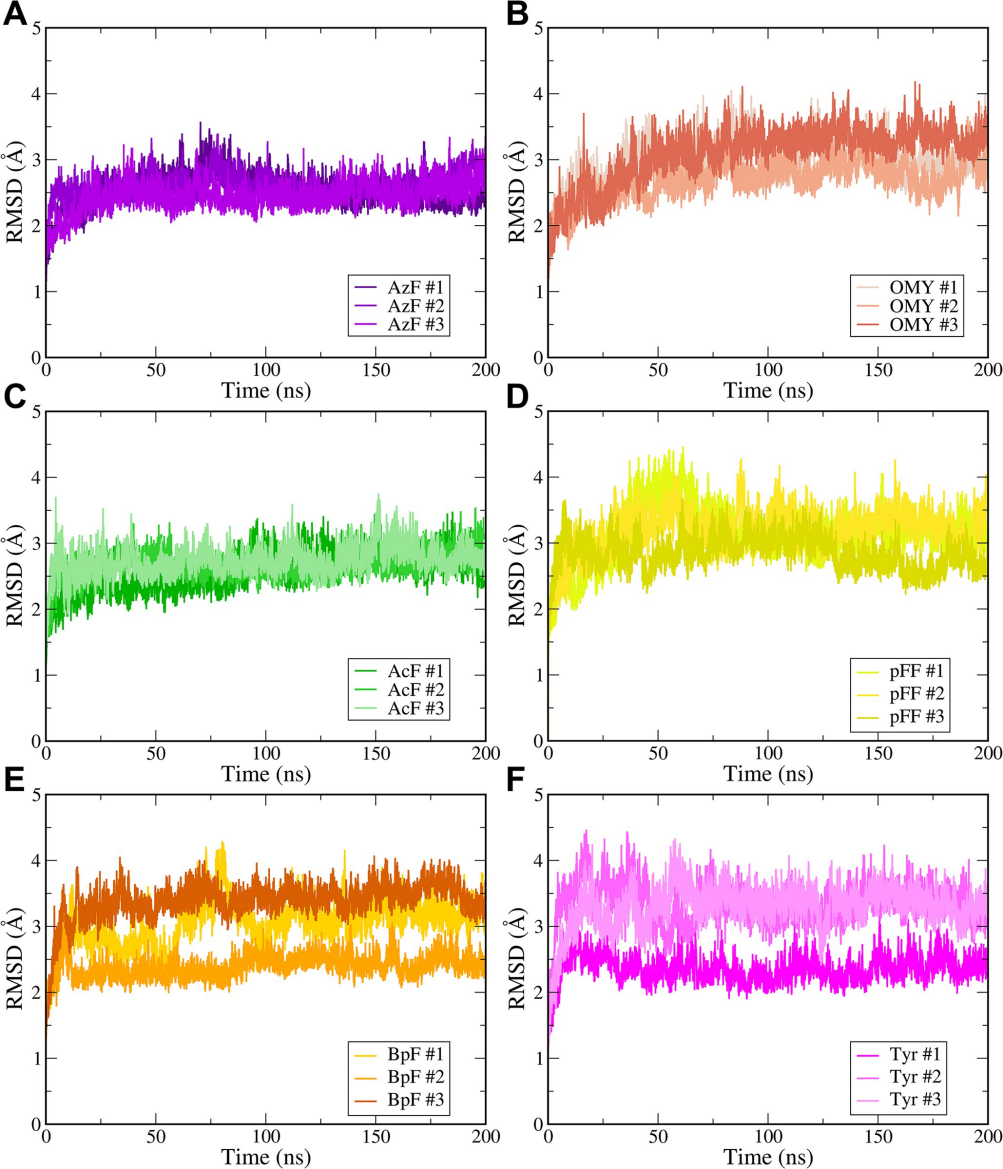
RMSD analysis of mutant aaRS and amino acid complexes in MD simulations.

The wild type *M*. *jannaschii* TyrRS activates with a dimeric structure to bind the tRNA, spanning the two subunits of the homodimer [19]. However, since the recognition of the amino acid involves only one subunit, MD simulations were performed using the monomer structure to elucidate the association between the active site of aaRS and the amino acid while reducing computational cost. MD simulations were carried out from the best docking poses to optimize the complex structures and demonstrate the binding properties. Simulations using five ncAAs and tyrosine were continued for 200 ns from the relaxed structure, with three simulation runs (Fig 4). Root-mean-square deviation (RMSD) compared to the starting frame was used to judge the conformational stability, which plateaued between 100 and 200 ns.

The binding free energy was calculated using the molecular mechanics/Poisson–Boltzmann surface area (MM/PBSA) method. The internal dielectric constant was set to 2 for moderately charged ligands [33]. Although entropy is crucial for determining absolute binding free energy, it was not calculated as it is not necessary for ranking the ligands [52]. Nonpolar solvation energy was calculated using two different methods. The first one was solvent accessible surface area (SASA) term method, where nonpolar solvation energy was estimated according to the SASA of solute. The other was the cavity and dispersion (CD) term method, where nonpolar solvation energy was divided into cavity and dispersion terms.

The binding free energies from three independent simulations were calculated using both the SASA term and CD term methods. The mean values are presented as mean ± pooled standard error of the mean in Tables 2 and 3, with detailed results provided in S2 Table. In Table 2, the total binding free energies for AzF and OMY were −20.6 and −20.2 kcal/mol, respectively, suggesting stronger binding affinities compared to AcF, pFF, and Tyr. However, BpF, despite not being a substrate of aaRS, exhibited a lower value of −22.2 kcal/mol. On the other hand, in Table 3, the total binding free energy calculated using the CD method showed −7.4 kcal/mol for the most favourable substrate AzF, and −5.4 kcal/mol for OMY. Tyr, the natural substrate used as a negative control for ncAA incorporation, showed a value of 0.4 kcal/mol. Other amino acids, which exhibited lower fluorescence similar to the negative control, ranged from −0.6 to 3.0 kcal/mol. A comparison between Tables 2 and 3 reveals differences only in polar and nonpolar solvation energy. The polar solvation energy difference between Tables 2 and 3 ranged from 1.4 to 6.3 kcal/mol due to variations in radii used for optimized calculations based on the nonpolar solvation method. This difference was minor compared to the energy difference of 15.7–24.6 kcal/mol in nonpolar solvation energy, highlighting the crucial role of the nonpolar solvation method in distinguishing between the results in Tables 2 and 3. Consequently, the total binding free energy calculated using the CD term showed good agreement with experimental results, demonstrating the substantial impact of the nonpolar solvation energy method on the total binding free energy, altering the ranking.

**Table 2:** Result of binding free energy calculated using the solvent accessible surface area (SASA) term method.

	ΔE_vdw_	ΔE_elec_	ΔG_polar_	ΔG_nonpolar_	ΔG_total_
AzF	−26.0 ± 0.5	−24.1 ± 0.7	33.4 ± 0.6	−3.9 ± 0.0	−20.6 ± 0.4
OMY	−25.4 ± 0.4	−21.4 ± 0.8	30.5 ± 0.7	−3.8 ± 0.0	−20.2 ± 0.4
AcF	−25.3 ± 0.5	−16.9 ± 1.0	29.5 ± 0.8	−4.0 ± 0.0	−16.8 ± 0.4
pFF	−18.0 ± 0.5	−18.9 ± 1.1	26.1 ± 1.0	−3.5 ± 0.0	−14.2 ± 0.4
BpF	−30.7 ± 0.6	−19.9 ± 1.5	33.0 ± 1.4	−4.6 ± 0.0	−22.2 ± 0.5
Tyr	−20.1 ± 0.6	−19.3 ± 0.9	28.6 ± 0.9	−3.5 ± 0.0	−14.4 ± 0.5

All values are reported in kcal/mol, and the energies are presented as pooled mean ± pooled standard error, calculated from triplicate simulations.

**Table 3:** Result of binding free energy calculated using the cavity and dispersion (CD) term method.

	ΔE_vdw_	ΔE_elec_	ΔG_polar_	ΔG_nonpolar_		ΔG_total_
				ΔG_cav_	ΔG_disp_	
AzF	−26.0 ± 0.5	−24.1 ± 0.7	27.1 ± 0.5	−20.3 ± 0.2	35.9 ± 0.2	−7.4 ± 0.6
OMY	−25.4 ± 0.4	−21.4 ± 0.8	26.5 ± 0.7	−19.3 ± 0.1	34.3 ± 0.2	−5.4 ± 0.5
AcF	−25.3 ± 0.5	−16.9 ± 1.0	28.1 ± 0.8	−19.3 ± 0.2	36.5 ± 0.2	3.0 ± 0.7
pFF	−18.0 ± 0.5	−18.9 ± 1.1	24.1 ± 0.9	−15.6 ± 0.2	27.8 ± 0.3	−0.6 ± 0.7
BpF	−30.7 ± 0.6	−19.9 ± 1.5	30.9 ± 1.3	−23.7 ± 0.3	43.7 ± 0.5	0.2 ± 0.7
Tyr	−20.1 ± 0.6	−19.3 ± 0.9	26.8 ± 0.9	−16.4 ± 0.3	29.5 ± 0.3	0.4 ± 0.8

All values are reported in kcal/mol, and the energies are presented as pooled mean ± pooled standard error, calculated from triplicate simulations.

In this study, the CD term method is more appropriate than the SADA term method because of the consistency with experimental results. However, the preference will be dependent on the study. The CD term method was devised for small molecules to address the limitations of the SASA model, which showed poor correlation with nonpolar organic molecules due to its orientation towards large solutes such as linear alkanes [35, 36]. A previous study reported that in systems where the ligand binds in a deep pocket, the CD model had a stronger correlation with the ligands binding at the centre of membrane proteins surrounded by amino acid residues [53]. Conversely, in the same study, the SASA model yielded better results with systems where ligands bind at the interface of proteins and lipids. Additionally, another study found that the CD model exhibited a smaller RMSD but a lower correlation of binding free energy compared to the SASA model [54]. Considering these studies and our results, the choice of method for calculating nonpolar solvation energy is not negligible for accurate calculations and proper ranking of ligands.

### Structure analysis

**Fig 5:**
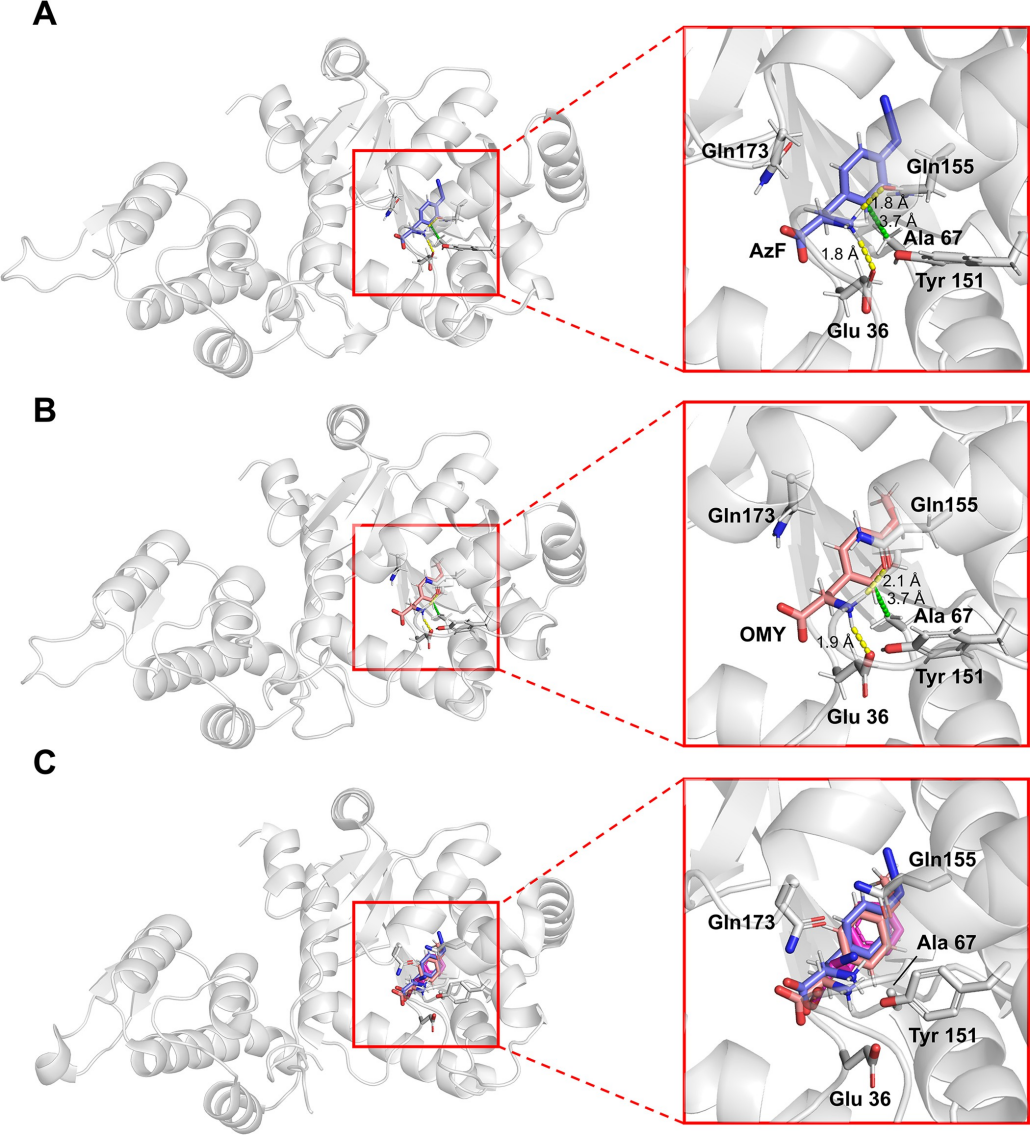
Representative structures of ligand–protein complexes in MD simulations. (A) AzF–aaRS complex. (B) OMY–aaRS complex. (C) Superimposed structure of AzF, OMY, and tyrosine on the crystal structure of *M*. *jannaschii* TyrRS (PDB 1J1U). The ligands AzF, OMY, and tyrosine are depicted in violet, salmon, and magenta, respectively. aaRS is shown in light grey. Hydrogen bonds and hydrophobic interactions are indicated by yellow and green dotted lines, respectively.

Recognition of amino acids by aaRS is achieved through non-covalent interactions such as hydrogen bonds, electrostatic (or salt bridge), hydrophobic, water-mediated, and π-interactions, which play an important role in the specific binding of ligands [55]. Representative structures of AzF–aaRS and OMY–aaRS complexes were obtained by clustering a 100–200 ns trajectories (Fig 5), and subsequently analysed using the PLIP tool except for hydrogen bonds. Hydrogen bonds were analysed using the cpptraj module with a 100–200 ns trajectories. The occupancy between the rotational hydrogen donor atom and the receptor atom was calculated by summing the specific occupancies, which were divided according to the specific hydrogens (Table 4). Full lists of hydrogen bond occupancy are provided in S3 Table.

**Table 4:** Hydrogen bond occupancies within the trajectory of 100–200 ns.

Acceptor	Donor	Fractions (AzF)	Fractions (OMY)
Glu36@O (F)	ncAA@N (A)	0.89 ± 0.10	0.83 ± 0.15
Gln155@O (F)	ncAA@N (A)	0.84 ± 0.03	0.72 ± 0.10
ncAA@O (C)	Gln173@N (F)	0.14 ± 0.07	0.08 ± 0.07
ncAA@O (C)	Glu36@N (F)	0.14 ± 0.13	0.01 ± 0.01

(A): amino group, (C): carboxyl group, (F): functional group

All values are presented as pooled mean ± standard deviation, calculated from triplicate simulations.

The AzF–aaRS and OMY–aaRS complex showed stable ligand binding, each showing the same interactions: one hydrophobic interaction and two hydrogen bonds (Table 4, Fig 5A and 5B). Ala67 hydrophobically interacted with the aromatic ring of ncAA, contributing to the recognition of the functional group. Gln155 formed a hydrogen bond with the amino group of ncAAs. These interactions were conserved from the wild type of *M*. *jannaschii* TyrRS and its complex with tyrosine [19]. Interestingly, Glu36 was involved in hydrogen bond formation, replacing Tyr151, which participated in recognizing the amino group of tyrosine in the *M*. *jannaschii* TyrRS–Tyr complex. The large functional groups of ncAAs caused the amino and carboxyl groups to be displaced outward from the pocket. This also made it more difficult for Gln173 to interact with the ncAAs, compared to its ability to form two hydrogen bonds with the amino and carboxyl groups of tyrosine in the wild-type TyrRS–Tyr complex, as indicated by the hydrogen bond occupancies (Table 4). The higher hydrogen bond occupancy of AzF compared to OMY contributed to the stable binding in the pocket, which was reflected in the lower electrostatic interaction energy shown in Table 3. Consequently, AzF was able to bind more stably at the active site than OMY.

## Conclusion

We conducted experimental studies on the expression of ncAA-incorporated sfGFP using five ncAAs and a mutant aaRS. The resulting differences in relative normalized fluorescence values were interpreted through computational studies, which included aaRS modelling, docking, MD simulation, and MM/PBSA. MM/PBSA was performed to calculate the binding free energies using two different nonpolar solvation methods (SASA term and CD term), leading to contrasting results depending on the method employed. The binding free energies calculated using the CD term method exhibited a stronger correlation with experimental results. Additionally, hydrogen bond and representative structure analyses confirmed that the recognition of amino groups significantly influenced ncAA binding. This study is expected to offer valuable insights into predicting the binding affinity of ncAAs, facilitating the rational design of aaRSs and the assessment of non-specific ncAA recognition due to aaRS polyspecificity.

## Supporting information

S1 Table Detailed docking results. (XLSX)

S2 Table Detailed values from MM/PBSA calculations. (XLSX)

S3 Table Hydrogen bond analysis of AzF and OMY. (XLSX)

## References

[1] Shandell MA, Tan Z, Cornish VW. Genetic Code Expansion: A Brief History and Perspective. Biochemistry. 2021;60(46):3455–69. doi: 10.1021/acs.biochem.1c00286 34196546 PMC8613843

[2] Dunkelmann DL, Oehm SB, Beattie AT, Chin JW. A 68-codon genetic code to incorporate four distinct non-canonical amino acids enabled by automated orthogonal mRNA design. Nature Chemistry. 2021;13(11):1110–7. doi: 10.1038/s41557-021-00764-5 34426682 PMC7612796

[3] Spinck M, Piedrafita C, Robertson WE, Elliott TS, Cervettini D, de la Torre D, et al. Genetically programmed cell-based synthesis of non-natural peptide and depsipeptide macrocycles. Nature Chemistry. 2023;15(1):61–9. doi: 10.1038/s41557-022-01082-0 36550233 PMC9836938

[4] Melnikov SV, Söll D. Aminoacyl-tRNA Synthetases and tRNAs for an Expanded Genetic Code: What Makes them Orthogonal? Int J Mol Sci. 2019;20(8). Epub 20190419. doi: 10.3390/ijms20081929 ; PubMed Central PMCID: PMC6515474. 31010123 PMC6515474

[5] Wang L, Brock A, Herberich B, Schultz PG. Expanding the genetic code of Escherichia coli. Science. 2001;292(5516):498–500. doi: 10.1126/science.1060077 . 11313494

[6] Blight SK, Larue RC, Mahapatra A, Longstaff DG, Chang E, Zhao G, et al. Direct charging of tRNACUA with pyrrolysine in vitro and in vivo. Nature. 2004;431(7006):333–5. doi: 10.1038/nature02895 15329732

[7] Neumann H, Peak-Chew SY, Chin JW. Genetically encoding Nε-acetyllysine in recombinant proteins. Nature Chemical Biology. 2008;4(4):232–4. doi: 10.1038/nchembio.73 18278036

[8] Lee D, Kim JG, Kim TW, Choi J-i. Development of orthogonal aminoacyl-tRNA synthetase mutant for incorporating a non-canonical amino acid. AMB Express. 2024;14(1):60. doi: 10.1186/s13568-024-01706-3 38782816 PMC11116331

[9] Beattie AT, Dunkelmann DL, Chin JW. Quintuply orthogonal pyrrolysyl-tRNA synthetase/tRNAPyl pairs. Nature Chemistry. 2023;15(7):948–59. doi: 10.1038/s41557-023-01232-y 37322102 PMC7615293

[10] Arranz-Gibert P, Patel JR, Isaacs FJ. The Role of Orthogonality in Genetic Code Expansion. Life (Basel). 2019;9(3). Epub 20190705. doi: 10.3390/life9030058 ; PubMed Central PMCID: PMC6789853. 31284384 PMC6789853

[11] Guo LT, Wang YS, Nakamura A, Eiler D, Kavran JM, Wong M, et al. Polyspecific pyrrolysyl-tRNA synthetases from directed evolution. Proc Natl Acad Sci U S A. 2014;111(47):16724–9. Epub 20141110. doi: 10.1073/pnas.1419737111 ; PubMed Central PMCID: PMC4250173. 25385624 PMC4250173

[12] Young DD, Young TS, Jahnz M, Ahmad I, Spraggon G, Schultz PG. An evolved aminoacyl-tRNA synthetase with atypical polysubstrate specificity. Biochemistry. 2011;50(11):1894–900. Epub 20110201. doi: 10.1021/bi101929e ; PubMed Central PMCID: PMC3694404. 21280675 PMC3694404

[13] Vargas-Rodriguez O, Sevostyanova A, Söll D, Crnković A. Upgrading aminoacyl-tRNA synthetases for genetic code expansion. Current Opinion in Chemical Biology. 2018;46:115–22. doi: 10.1016/j.cbpa.2018.07.014 30059834 PMC6214156

[14] Zhang D, Vaidehi N, Goddard WA 3rd, Danzer JF, Debe D. Structure-based design of mutant Methanococcus jannaschii tyrosyl-tRNA synthetase for incorporation of O-methyl-L-tyrosine. Proc Natl Acad Sci U S A. 2002;99(10):6579–84. doi: 10.1073/pnas.052150499 ; PubMed Central PMCID: PMC124445. 12011422 PMC124445

[15] Sun R, Zheng H, Fang Z, Yao W. Rational design of aminoacyl-tRNA synthetase specific for p-acetyl-l-phenylalanine. Biochemical and Biophysical Research Communications. 2010;391(1):709–15. doi: 10.1016/j.bbrc.2009.11.125 19944076

[16] Ren W, Truong TM, Ai H-w. Study of the Binding Energies between Unnatural Amino Acids and Engineered Orthogonal Tyrosyl-tRNA Synthetases. Scientific Reports. 2015;5(1):12632. doi: 10.1038/srep12632 26220470 PMC4518261

[17] Lee D, Kim M-K, Choi J-i. Development of Orthogonal Aminoacyl tRNA Synthetase Mutant with Enhanced Incorporation Ability with Para-azido-L-phenylalanine. Biotechnol Bioprocess Eng. 2023. doi: 10.1007/s12257-022-0252-0

[18] Waterhouse A, Bertoni M, Bienert S, Studer G, Tauriello G, Gumienny R, et al. SWISS-MODEL: homology modelling of protein structures and complexes. Nucleic Acids Res. 2018;46(W1):W296–w303. doi: 10.1093/nar/gky427 ; PubMed Central PMCID: PMC6030848. 29788355 PMC6030848

[19] Kobayashi T, Nureki O, Ishitani R, Yaremchuk A, Tukalo M, Cusack S, et al. Structural basis for orthogonal tRNA specificities of tyrosyl-tRNA synthetases for genetic code expansion. Nat Struct Biol. 2003;10(6):425–32. doi: 10.1038/nsb934 . 12754495

[20] Chin JW, Santoro SW, Martin AB, King DS, Wang L, Schultz PG. Addition of p-Azido-l-phenylalanine to the Genetic Code of Escherichia coli. Journal of the American Chemical Society. 2002;124(31):9026–7. doi: 10.1021/ja027007w 12148987

[21] Eberhardt J, Santos-Martins D, Tillack AF, Forli S. AutoDock Vina 1.2.0: New Docking Methods, Expanded Force Field, and Python Bindings. Journal of Chemical Information and Modeling. 2021;61(8):3891–8. doi: 10.1021/acs.jcim.1c00203 34278794 PMC10683950

[22] Kim S, Chen J, Cheng T, Gindulyte A, He J, He S, et al. PubChem 2023 update. Nucleic Acids Res. 2022;51(D1):D1373–D80. doi: 10.1093/nar/gkac956 36305812 PMC9825602

[23] Gordon JC, Myers JB, Folta T, Shoja V, Heath LS, Onufriev A. H++: a server for estimating pKas and adding missing hydrogens to macromolecules. Nucleic Acids Res. 2005;33(Web Server issue):W368–71. doi: 10.1093/nar/gki464 ; PubMed Central PMCID: PMC1160225. 15980491 PMC1160225

[24] Maier JA, Martinez C, Kasavajhala K, Wickstrom L, Hauser KE, Simmerling C. ff14SB: Improving the Accuracy of Protein Side Chain and Backbone Parameters from ff99SB. Journal of Chemical Theory and Computation. 2015;11(8):3696–713. doi: 10.1021/acs.jctc.5b00255 26574453 PMC4821407

[25] He X, Man VH, Yang W, Lee T-S, Wang J. A fast and high-quality charge model for the next generation general AMBER force field. The Journal of Chemical Physics. 2020;153(11). doi: 10.1063/5.0019056 32962378 PMC7728379

[26] Pieffet G, Petukhov PA. Parameterization of aromatic azido groups: application as photoaffinity probes in molecular dynamics studies. J Mol Model. 2009;15(11):1291–7. Epub 20090314. doi: 10.1007/s00894-009-0488-z ; PubMed Central PMCID: PMC2745498. 19288146 PMC2745498

[27] Rizzuti B. Molecular simulations of proteins: From simplified physical interactions to complex biological phenomena. Biochimica et Biophysica Acta (BBA)—Proteins and Proteomics. 2022;1870(3):140757. doi: 10.1016/j.bbapap.2022.140757 35051666

[28] Jorgensen WL, Chandrasekhar J, Madura JD, Impey RW, Klein ML. Comparison of simple potential functions for simulating liquid water. The Journal of Chemical Physics. 1983;79(2):926–35. doi: 10.1063/1.445869

[29] Case HMA D.A., Belfon K., Ben-Shalom I.Y., Berryman J.T., Brozell S.R., Cerutti D.S., Amber 2023. University of California, San Francisco. 2023.

[30] Hopkins CW, Le Grand S, Walker RC, Roitberg AE. Long-Time-Step Molecular Dynamics through Hydrogen Mass Repartitioning. Journal of Chemical Theory and Computation. 2015;11(4):1864–74. doi: 10.1021/ct5010406 26574392

[31] Miller BR III, McGee TD, Jr., Swails JM, Homeyer N, Gohlke H, Roitberg AE. MMPBSA.py: An Efficient Program for End-State Free Energy Calculations. Journal of Chemical Theory and Computation. 2012;8(9):3314–21. doi: 10.1021/ct300418h 26605738

[32] Case DA, Aktulga HM, Belfon K, Cerutti DS, Cisneros GA, Cruzeiro VWD, et al. AmberTools. Journal of Chemical Information and Modeling. 2023;63(20):6183–91. doi: 10.1021/acs.jcim.3c01153 37805934 PMC10598796

[33] Sun H, Li Y, Shen M, Tian S, Xu L, Pan P, et al. Assessing the performance of MM/PBSA and MM/GBSA methods. 5. Improved docking performance using high solute dielectric constant MM/GBSA and MM/PBSA rescoring. Physical Chemistry Chemical Physics. 2014;16(40):22035–45. doi: 10.1039/c4cp03179b 25205360

[34] Akkus E, Tayfuroglu O, Yildiz M, Kocak A. Revisiting MMPBSA by Adoption of MC-Based Surface Area/Volume, ANI-ML Potentials, and Two-Valued Interior Dielectric Constant. J Phys Chem B. 2023;127(20):4415–29. Epub 20230512. doi: 10.1021/acs.jpcb.3c00834 ; PubMed Central PMCID: PMC10226125. 37171911 PMC10226125

[35] Tan C, Tan Y-H, Luo R. Implicit Nonpolar Solvent Models. The Journal of Physical Chemistry B. 2007;111(42):12263–74. doi: 10.1021/jp073399n 17918880

[36] Sitkoff D, Sharp KA, Honig B. Accurate calculation of hydration free energies using macroscopic solvent models. The Journal of Physical Chemistry. 1994;98(7):1978–88.

[37] Tan C, Yang L, Luo R. How well does Poisson-Boltzmann implicit solvent agree with explicit solvent? A quantitative analysis. J Phys Chem B. 2006;110(37):18680–7. doi: 10.1021/jp063479b . 16970499

[38] Roe DR, Cheatham TE, III. PTRAJ and CPPTRAJ: Software for Processing and Analysis of Molecular Dynamics Trajectory Data. Journal of Chemical Theory and Computation. 2013;9(7):3084–95. doi: 10.1021/ct400341p 26583988

[39] Salentin S, Schreiber S, Haupt VJ, Adasme MF, Schroeder M. PLIP: fully automated protein-ligand interaction profiler. Nucleic Acids Res. 2015;43(W1):W443–7. Epub 20150414. doi: 10.1093/nar/gkv315 ; PubMed Central PMCID: PMC4489249. 25873628 PMC4489249

[40] Schrodinger, LLC. The PyMOL Molecular Graphics System, Version 3.0.0. 2010.

[41] Francklyn CS, First EA, Perona JJ, Hou YM. Methods for kinetic and thermodynamic analysis of aminoacyl-tRNA synthetases. Methods. 2008;44(2):100–18. doi: 10.1016/j.ymeth.2007.09.007 ; PubMed Central PMCID: PMC2288706. 18241792 PMC2288706

[42] Cho J, Lim SI, Yang BS, Hahn YS, Kwon I. Generation of therapeutic protein variants with the human serum albumin binding capacity via site-specific fatty acid conjugation. Scientific Reports. 2017;7(1):18041. doi: 10.1038/s41598-017-18029-y 29269881 PMC5740134

[43] Bird RE, Lemmel SA, Yu X, Zhou QA. Bioorthogonal Chemistry and Its Applications. Bioconjugate Chemistry. 2021;32(12):2457–79. doi: 10.1021/acs.bioconjchem.1c00461 34846126

[44] Kölmel DK, Kool ET. Oximes and Hydrazones in Bioconjugation: Mechanism and Catalysis. Chem Rev. 2017;117(15):10358–76. Epub 20170622. doi: 10.1021/acs.chemrev.7b00090 ; PubMed Central PMCID: PMC5580355. 28640998 PMC5580355

[45] Chin JW, Martin AB, King DS, Wang L, Schultz PG. Addition of a photocrosslinking amino acid to the genetic code of Escherichia coli. Proceedings of the National Academy of Sciences. 2002;99(17):11020–4. doi: 10.1073/pnas.172226299 12154230 PMC123203

[46] Brüninghoff K, Wulff S, Dörner W, Geiss-Friedlander R, Mootz HD. A Photo-Crosslinking Approach to Identify Class II SUMO-1 Binders. Front Chem. 2022;10:900989. Epub 20220530. doi: 10.3389/fchem.2022.900989 ; PubMed Central PMCID: PMC9191277. 35707458 PMC9191277

[47] Ishiwata K, Tsukada H, Kubota K, Nariai T, Harada N, Kawamura K, et al. Preclinical and clinical evaluation of O-[11C]methyl-L-tyrosine for tumor imaging by positron emission tomography. Nucl Med Biol. 2005;32(3):253–62. doi: 10.1016/j.nucmedbio.2004.11.005 . 15820760

[48] Budisa N, Wenger W, Wiltschi B. Residue-specific global fluorination of Candida antarctica lipase B in Pichia pastoris. Molecular BioSystems. 2010;6(9):1630–9. doi: 10.1039/c002256j 20431819

[49] Chatterjee A, Sun SB, Furman JL, Xiao H, Schultz PG. A Versatile Platform for Single- and Multiple-Unnatural Amino Acid Mutagenesis in Escherichia coli. Biochemistry. 2013;52(10):1828–37. doi: 10.1021/bi4000244 23379331 PMC3855549

[50] Koch NG, Goettig P, Rappsilber J, Budisa N. Engineering Pyrrolysyl-tRNA Synthetase for the Incorporation of Non-Canonical Amino Acids with Smaller Side Chains. Int J Mol Sci. 2021;22(20). Epub 20211017. doi: 10.3390/ijms222011194 ; PubMed Central PMCID: PMC8538471. 34681855 PMC8538471

[51] Young TS, Ahmad I, Yin JA, Schultz PG. An enhanced system for unnatural amino acid mutagenesis in E. coli. J Mol Biol. 2010;395(2):361–74. Epub 2009/10/27. doi: 10.1016/j.jmb.2009.10.030 . 19852970

[52] Hou T, Wang J, Li Y, Wang W. Assessing the Performance of the MM/PBSA and MM/GBSA Methods. 1. The Accuracy of Binding Free Energy Calculations Based on Molecular Dynamics Simulations. Journal of Chemical Information and Modeling. 2011;51(1):69–82. doi: 10.1021/ci100275a 21117705 PMC3029230

[53] Wang S, Sun X, Cui W, Yuan S. MM/PB(GB)SA benchmarks on soluble proteins and membrane proteins. Front Pharmacol. 2022;13:1018351. Epub 20221201. doi: 10.3389/fphar.2022.1018351 ; PubMed Central PMCID: PMC9751045. 36532746 PMC9751045

[54] Wang C, Nguyen PH, Pham K, Huynh D, Le T-BN, Wang H, et al. Calculating protein–ligand binding affinities with MMPBSA: Method and error analysis. Journal of Computational Chemistry. 2016;37(27):2436–46. doi: 10.1002/jcc.24467 27510546 PMC5018451

[55] Kaiser F, Krautwurst S, Salentin S, Haupt VJ, Leberecht C, Bittrich S, et al. The structural basis of the genetic code: amino acid recognition by aminoacyl-tRNA synthetases. Scientific Reports. 2020;10(1):12647. doi: 10.1038/s41598-020-69100-0 32724042 PMC7387524

